# The Moderating Roles of Sensation Seeking and Worry among Nature-Based Adventure Tourists

**DOI:** 10.3390/ijerph18042021

**Published:** 2021-02-19

**Authors:** Kiattipoom Kiatkawsin, Ngoc Anh Bui, Richard Hrankai, Kwangmin Jeong

**Affiliations:** 1 Tourism Industry Data Analytics Lab (TIDAL), Department of Hospitality and Tourism Management, Sejong University, Seoul 05006, Korea; kiatkawsin@gmail.com; 2 Department of Hospitality and Tourism Management, Sejong University, Seoul 05006, Korea; ngocanhbui.bna@gmail.com; 3 Department of Tourism and Service Management, MODUL University Vienna, 1190 Vienna, Austria; richard.hrankai@modul.ac.at; 4 Tourism Industry Research Division, Korea Culture & Tourism Institute, Seoul 07511, Korea

**Keywords:** adventure tourism, Vietnam tourism, theory of planned behavior, sensation seeking, worry, involvement

## Abstract

The adventure tourism subsector continues to be popular today. Both industry and academia define adventure tourism’s scope from either the physical (e.g., outdoor activity and physical activity) or psychological aspects (e.g., thrill seeking and challenges). Recent studies have pointed out that adventure tourism can be interpreted differently across cultures and markets. Still, risk has always been universally accepted as an essential characteristic of adventure tourism. Thus, most empirical research has studied the role of risk as one of the motivations. However, attempts to investigate related elements that are either a response to or a coping mechanism for the presence of risk are scarce. This present study adopted one of the most prominent frameworks in explaining behavioral intentions, the theory of planned behavior, and included involvement and knowledge variables to extend it. Furthermore, the sensation-seeking and worry constructs were tested for their moderating impact on intentions to participate in adventure tours. The results of structural equation modeling and multigroup invariance tests revealed that subjective norms were not a significant predictor of intentions, while both sensation seeking and worry significantly moderated the relationships between the study variables.

## 1. Introduction

Adventure activities have been studied in the tourism context since the early days of tourism research [1,2,3,4]. Today, the popularity of the adventure tourism subsector remains relatively strong [5,6,7]. Scholars have attributed this sector’s continuous growth to the “commodification” of adventure tourism [2,5]. Commodification refers to how adventure tourism activities were perceived as predominantly high-risk, high-difficulty activities usually reserved for those with advanced skills, and an intense commitment to include more leisure activities suitable for the mass market [1,2,8]. Specifically, the commodification process involves three characteristics. The first includes how the experience’s adventure element has been choreographed and packaged to be more accessible by the adventure tour guide [5]. Secondly, the risk that is typically associated with adventure activities has been diluted to also include perceived risk as well as actual risks [2]. Thirdly, the expanded definition of adventure tourism typically consists of the natural environment, making many nature-based activities considered a part of adventure tourism [3,9].

Despite the varying definitions and ambiguous categorization of adventure tourism, several elements remain consistent and still vital for the characteristics of adventure tours [4,7]. The Adventure Travel Trade Association (ATTA)’s definition of adventure tourism states that at least two of the following three elements must be included: physical activity, the natural environment, and cultural immersion [7]. Another popular definition by Buckley (2007) stated that adventure tours generally include outdoor activities featuring the natural terrain that are exciting for tourists and have an economic benefit for experience providers [10]. Moreover, the notion of “risk” involved in the activities remains one of the most critical elements associated with adventure, and to some degree, it may be the cause of the inconsistency in the scope and definition of adventure tourism [1,4,7,11]. Specifically, the soft–hard classification was predominantly based on the level of risk and danger associated with an activity. Therefore, soft adventure tours can include bird watching and camping in a park, while hard adventure tours include mountaineering or flights in space [4,7]. Still, both soft and hard adventure tours still fall within the same umbrella term of adventure tourism but are motivated differently [3,12].

The role of risk and danger has been studied extensively in the tourism context because of its significance in determining adventure tourism experiences. Notably, risk has been used to classify activities into soft or hard adventure [1,2,6,13,14], determine the types of motivation for participating in adventure activities [1,6,10,15,16], and determine experience quality and post-trip evaluation and behaviors [6,17,18,19,20,21,22]. Furthermore, previous researchers have studied other concepts that are still related to risks, such as a sense of accomplishment [23], the use of specific skills [24], the opportunity to challenge one’s physical and mental limits [15], and the chance to learn new skills and discover oneself [7,25]. Nevertheless, studies focusing on the coping mechanism and the preparation in the pre-trip stage, especially in relation to the attitudinal dimensions of adventure tourists, are still lacking. Previous experience and existing knowledge related to adventure activities were found to have significant influences on attitude [6,23,26,27]. Thus, attitude helps to determine the likelihood of participation in the activity [28,29,30,31,32]. Moreover, constructs such as sensation seeking and worry have been found to help in coping with the level of perceived risks usually associated with adventure tours [33,34,35,36,37].

This present study identified two dimensions that are critical determinants of the attitudinal perception of adventure activities, namely, involvement and knowledge. Involvement refers to the level of interest, emotional attachment, state of motivation, and perceived importance of a topic [38,39,40,41]. Individuals possessing a high level of involvement in a given subject were found to be highly active in reading and sharing opinions online, have a favorable and positive attitude about the topic, and ultimately be able to accumulate a large amount of experience and knowledge of the subject [26,38,39,40]. Consequently, the tourists’ knowledge or experience has been found to be a critical determinant of attitude, risk perception, and purchase/visit intentions [42,43,44,45]. The study adopted one of the most popular frameworks used for predicting future behaviors that are predominantly based on individuals’ cognition and attitude, the theory of planned behavior (TPB) [46,47,48,49]. Thus, the study attempted to extend the TPB with the addition of involvement and knowledge.

Furthermore, two personality traits, sensation seeking and worry, have been identified as potential moderators of the study constructs’ relationships. Sensation seeking refers to an individual’s need for varied, novel, and complex sensations [33,34]. Psychologists found that individuals high in sensation-seeking characteristics are often attracted to high-risk activities, such as adventure activities [6,28,35]. On the other hand, worry is related to the constant cognitive thinking about the future’s uncertain outcomes [36]. Both sensation seeking and worry can be important moderators for the other proposed relationships, as both constructs are related to the tourist’s perceived risk. For example, high sensation seekers tend to perceive risky behaviors as less risky than low sensation seekers, thus being likely to engage in such activities more often [35]. Meanwhile, people worry because they believe it can minimize adverse outcomes and encourage better preparation [36].

The roles and relationships of involvement, knowledge, sensation seeking, and worry have not been well established in tourist behavior research, especially in the adventure tourism context. Thus, this present study aimed to extend the TPB with four new constructs and validate the proposed conceptual model by sampling Vietnamese nature-based adventure tourists. The following objectives were developed to help achieve the research aim. Objective one was to propose a conceptual framework that extended the TPB with the involvement and knowledge constructs. Then, objective two was to validate the conceptual model using samples of Vietnamese adventure tourists. Lastly, objective three was to test the moderating roles of sensation seeking and worry using the multigroup invariance test.

## 2. Literature Review

### 2.1. Involvement and Knowledge

The concept of involvement, also referred to as consumer involvement, has often been defined using Zaichkowsky’s (1985) definition as “a person’s perceived relevance of the object based on inherent needs, values, and interests” [50]. Other researchers have also included the perceived importance of a product to the scope of involvement [38]. Furthermore, another group of studies demonstrated that emotional attachment or arousal is also an important element of involvement [39,51]. Involvement is considered an unobservable state of motivation, interests, and arousal [51,52]. The concept refers to both the mental state and the behaviors, making it an essential construct in consumer behavior studies due to its efficacy in predicting both attitude and behaviors [39,53]. In other words, involvement can refer to the level of personal attachment or engagement a person has to a brand or an activity, such as for those who enjoy traveling or dining [49,52]. At the same time, involvement can refer to how often a person engages in a behavior, such as frequent travelers traveling more often than the average person or those who read and write travel reviews online often being more involved in trip planning [26,39,40,54].

The critical role of involvement is even more pronounced among high-involvement products. High-involvement products typically require more research and complex decision-making processes, have relatively high costs, and are not often purchased, such as luxury goods, banking service providers, insurance, and travel decisions [26,40,55]. Moreover, some challenging adventure activities usually require the mastering of skills by the participants [7]. The result of involvement is closely associated with the amount of knowledge or experience an individual accumulates [42]. Hence, involvement and knowledge are often studied together [26,56].

Knowledge, in this context, is borrowed from the concept of consumer knowledge [42]. It refers to the assumption that consumers have some experience with information about the product [43]. In other words, when a tourist has knowledge about adventure tourism, he or she is assumed to have prior experience with the activities. The same concept has also been referred to as familiarity or subjective knowledge [29,57]. Having knowledge of adventure activities is crucial because it can help to reduce both perceived and actual risks [7,43]. More importantly, an expert consumer is likely to have superior knowledge as a result of a high level of involvement and commitment. Therefore, the study proposed a significant relationship between involvement and knowledge, as reflected in Hypothesis 1.

**Hypothesis** **1.** *Involvement significantly impacts knowledge among adventure tourists.*

### 2.2. Theory of Planned Behavior

The theory of planned behavior remains a popular framework for predicting human behaviors today [58,59,60]. Its application can be found in diverse disciplines with a great degree of success, from predicting young people’s attitudes towards texting while driving to farmers’ intentions to use chemical fertilizers [61,62]. In tourism studies, the TPB has been effective in predicting behavioral intentions in several contexts, such as volunteer tourism, decisions to travel to Tibet, intentions to visit Cuba, bicycle tour participation, and many more [48,58,59,60]. The TPB has not only been a widely accepted model in its original form but formed the basis for theory extensions. For example, the model of goal-directed behavior was developed as an effort for deepening and broadening the TPB [6,63]. The TPB was also successfully merged with other frameworks to improve its effectiveness in specific fields such as environmentally responsible behaviors and other tourist behavioral research projects [6,49,59,62,64].

The TPB’s development was based on consumer attitudes [46,47]. Attitude has been found to be one of the most reliable and robust proxies of behavior, as consumers are unlikely to engage in an activity or purchase a product that they have a negative attitude toward [65,66]. Attitudes are either favorable/positive or unfavorable/negative beliefs someone holds about a product, a brand, an activity, or a destination [28,29,62]. In the context of this research project, the attitude construct measures the level of favorable (or unfavorable) evaluation a person has for adventure tourism activities. The TPB also consists of two other variables, subjective norms and perceived behavioral control. Subjective norms refer to the beliefs individuals hold regarding how much the people around them, such as friends and family, would support the activity in question [6,66]. In practice, subjective norms measure whether friends and family approve or disapprove of their planned intention or their intention to participate in an adventure tour in this case. Perceived behavioral control is the held belief individuals have regarding their ability to participate in the action [64]. In this case, the ability can be the amount of resources (such as money) required to participate, the necessary skill and capability, and their confidence to partake [67]. The three variables then predict behavioral intentions, which has received comprehensive support for being an accurate predictor of actual behavior [62].

The consequences of involvement and knowledge have been well established by previous studies. A study found that the intention to revisit the same hotel brand was influenced by the relatively high level of involvement, as involvement implies the likelihood of the guests to read and interact with the brands’ social media accounts. Thus, they are continually being reminded of the brand and their previous experience. Eventually, their awareness and familiarity propel them to choose the same hotel for their next travel [53]. Another study about food and travel found attitude to be included by the multidimensional concept of food-related knowledge, and internal attitude significantly impacted behavioral intentions [49]. Moreover, perceived control has also been studied as a consequence of prior experience and familiarity. In an adventure tourism context, the relationship between perceived control and intentions was completely mediated by the desire to visit an adventure destination [6]. A research project on travelers’ perceived risk of the Middle East also found empirical support for how tourists’ previous experiences influenced knowledge and, eventually, intentions to plan for a trip. More importantly, the study also found that the perceived risks were lower, as tourists have accumulated more knowledge about the Middle East [42]. Thus, the hierarchical flow from involvement to knowledge, knowledge to attitude and perceived control, and intentions has been empirically supported. The following hypotheses were consequently proposed.

**Hypothesis** **2.** *Involvement significantly impacts attitude among adventure tourists.*

**Hypothesis** **3.** *Involvement significantly impacts perceived behavioral control among adventure tourists.*

**Hypothesis** **4.** *Knowledge significantly impacts attitude among adventure tourists.*

**Hypothesis** **5.** *Knowledge significantly impacts perceived behavioral control among adventure tourists.*

**Hypothesis** **6.** *Attitude significantly impacts behavioral intentions among adventure tourists.*

**Hypothesis** **7.** *Subjective norms significantly impact behavioral intentions among adventure tourists.*

**Hypothesis** **8.** *Perceived behavioral control significantly impacts behavioral intentions among adventure tourists.*

### 2.3. Sensation Seeking

Risks may have often been perceived as something to avoid or minimize from the perspective of tourists looking for activities to attend and destinations to visit [28,60]. However, in the context of adventure tourism, risks may be the appeal that pulls tourists to the destination [15,36]. By definition, adventure tourism involves tourists voluntarily seeking to engage in high-risk activities driven by various motives such as challenging themselves, gaining novel experience, exploring their physical and mental limits, discovering new experiences, and more [6,28,36]. However, limited research has examined risk as a favorable attitudinal perception and as a motivation for participating in an activity. This present study identified sensation-seeking characteristics to help explain the relationships among the study variables.

Sensation seeking is a personality trait that determines the extent to which individuals like to seek novel experiences in their lives. Those exhibiting high sensation-seeking behaviors are likely to seek experiences that offer arousal stimulation, novelty compared to the ordinary, or a combination of the two [33,34]. Adventure activities or other high-risk activities were found to be applicable to satisfy those sensation seekers [6,28,35]. The context of adventure tourism not only comprises risky physical activities but also includes cultural immersion and the travel element, making the concept of sensation seeking more applicable than other related concepts such as risk/thrill seeking, rush, edgework, and flow [3,5,7]. A previous study found that those with low sensation seeking struggled to cope with stress more than high sensation seeking individuals [68]. Additionally, sensation seeking was found to moderate the relationship between attitude and behavior among young people in the leisure context [69]. In the same vein, this study posits that sensation seeking moderates the relationships between the proposed study variables as reflected in the following hypotheses.

**Hypothesis** **9.** *Sensation seeking significantly moderates the relationships between (a) involvement and knowledge, (b) involvement and attitude, (c) involvement and perceived behavioral control, (d) knowledge and attitude, (e) knowledge and perceived behavioral control, (f) attitude and behavioral intentions, (g) subjective norms and behavioral intentions, (h) perceived behavioral control and behavioral intentions.*

### 2.4. Worry

Worry is a predominantly cognitive construct that describes an activity involving a consistent thought related to future events [36]. In other words, worry is believed to be caused by individuals’ evaluation of future events’ uncertain outcomes. Because the outcomes can be ambiguous or uncertain, those who require explicit and predictable evidence in their decision-making processes tend to struggle to cope [37,70]. Thus, they often start a chain of thoughts that revolves around possible outcomes, which are unknown [36]. Risky activities such as adventure tours imply a generally higher chance of the participants sustaining injuries or any other undesirable effects [7,28,38,42,43]. Hence, the concepts of risk, uncertainty, and worry are inevitably linked.

**Figure 1:**
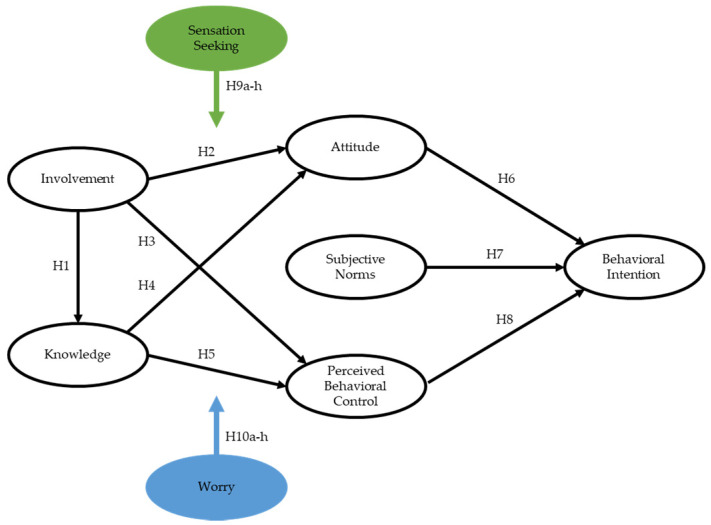
Proposed conceptual model.

Although worrying is widely accepted to be a negative experience because most of the thoughts are associated with negative outcomes and unpleasant effects, people still believe that worry can be helpful even if they also accept that worrying is ultimately pointless [36,37]. Most notably, worry is linked to self-protective behaviors. In other words, worriers tend to be more thorough when planning and are likely to engage in more preparations and follow safety precautions more precisely [28,42,43,70]. Prior knowledge and experience play a significant role in aiding the decision-making process for those who worry because they can anticipate the type of risk and have more information on preparation and cautionary behaviors [43,70]. Consequently, the level of worry should either strengthen or weaken the relationships between the study variables. For example, those with a lower level of worry may hold a more favorable attitude towards adventure tourism, as they think about the uncertain outcomes less than those with a high level of worry. The following hypotheses were developed to test the moderating role of worry. Additionally, Figure 1 graphically illustrates the study variables and the relationships among them.

**Hypothesis** **10.** *Worry significantly moderates the relationships between (a) involvement and knowledge, (b) involvement and attitude, (c) involvement and perceived behavioral control, (d) knowledge and attitude, (e) knowledge and perceived behavioral control, (f) attitude and behavioral intentions, (g) subjective norms and behavioral intentions, (h) perceived behavioral control and behavioral intentions.*

## 3. Methods

### 3.1. Measurement Items

This present study adopted all the measurement items from previous studies. The eight latent constructs in the proposed conceptual model were all measured using a total of 37 items. The three antecedents of the TPB—attitude, subjective norms, and perceived behavioral control—were measured using items adopted from Lee et al. (2012) [67]. The knowledge construct was measured using three items from Algesheimer et al. (2005) [71]. Four items from Amaro and Duarte (2015) were adopted to measure the involvement construct [39]. The measurement items used to measure the two moderating variables, sensation seeking and worry, were adopted from Hoyle et al. (2002) and Larsen et al. (2009), respectively. Lastly, the dependent variable, behavioral intentions, was measured using four items from Lee at al. (2012) [67]. After adoption, the wording of the original items was adapted to accurately reflect the study’s adventure tourism context while still maintaining the original meaning of the items. Moreover, 5-point Likert-type scales were used to measure all the measurement items. A list of all the measurement items used in the survey can be seen in Table A1 in the Appendix A. 

### 3.2. Survey Development

The survey also included a short cover letter that briefly described the nature of the research and explained the research contexts to the participants. Statements assuring the anonymity and noncommercial use of the data were provided in this section. Two qualifying questions were then added after the cover letter. The questions asked if the participant had experienced adventure tours in the past two years or planned to partake in an adventure tour within the next two years. Only those who answered at least one of the two qualifying questions were asked to complete the rest of the survey. In the last section of the survey, a number of demographic and travel characteristic questions were added. The draft version of the survey was then subjected to a pre-test. Subsequently, the final English version of the survey was translated into Vietnamese. The translated version was distributed to Vietnamese travelers for another round of pre-testing. Pre-testers recommended including a definition of adventure tourism and providing examples of popular adventure tour destinations and activities within Vietnam.

### 3.3. Sampling and Data Collection

The research project sampled any Vietnamese tourists who had participated in an adventure tour within the last two years and those who had yet to participate but were planning to partake within the next two years. In order to reach those groups of adventure tourists, the final version of the survey in Vietnamese was input into a Google Form. The link to the survey was then distributed across various travel forums, online communities, and social network groups related to adventure tourism. Physical copies of the survey were also distributed to a smaller group of known adventure tourists to supplement the online collection. Due to the niche interests of adventure tourism, a mixture of snowball and convenience sampling techniques was deployed.

### 3.4. Data Screening and Sample Profiles

A total of 308 completed surveys were collected. Both the online data and offline collection were combined into a single file using the IBM SPSS software version 23. The raw data were subjected to data screening for cases with many missing data, unengaged responses, normality, and outliers. The raw data contained no missing data, and the screening process found no evidence of outliers. Unengaged responses were identified by calculating the standard deviation for each row. Cases with a standard deviation lower than 0.5 were thoroughly examined. In total, 21 cases were removed due to suspected unengagement. The remaining 287 cases’ distribution fell within the acceptable range. Specifically, the skewness ranged from −1.732 to −0.542, and the kurtosis ranged from −0.818 to 6.421.

Among the 287 retained for further analysis, 53.3% were male, and 46.3% were female. Slightly over half, at 51.2%, reported their age to be between 27 and 35 years old. Ages ranging between 23 and 26 years old represented 25.4% of the research samples. Only 4.9% of the samples reported being older than 45 years old. The majority were single (59.9%), followed by married (33.1%). Most of the samples held university degrees; bachelor’s degrees accounted for 55.1%, and master’s degree holders accounted for 22.3%. Additionally, most of the sample were either full-time employees (54.0%) or self-employed (23.0%). Students accounted for 16.7% of the samples.

The research samples reported their previous experience with adventure tours. The largest group (39.7%) had participated in an adventure tour at least three times, while 33.1% had never taken one before. Among those who had been or were planning to go, friends were the most popular (63.8%) accompanying persons. Going alone accounted for 10.8%, while going with family accounted for 12.2%. Lastly, mountain climbing was the most popular activity (39.7%) among the research samples, followed by trekking (22.6%), cave exploring (20.6%), waterfall diving (7.3%), and other activities (9.8%). 

## 4. Results

### 4.1. Confirmatory Factor Analysis

The hypothesis testing followed the two-step structural equation modeling (SEM) approach suggested by Anderson and Gerbing (1988) [72]. The first step was the confirmatory factor analysis (CFA), which focused on the relationship between the observable items and the latent construct, or the measurement model. The first step examined the model fit statistics between the study variables and the data. The results yielded satisfactory model fit scores (*χ^2^* = 466.792, *df* = 296, *p* = < 0.001, *χ*^2^/*df* = 1.577, RMSEA = 0.045, CFI = 0.971, and IFI = 0.971). Next, composite reliability (CR) scores were calculated for each latent construct. The scores ranged between 0.813 and 0.940, all higher than the minimum threshold of 0.7 [73]. Hence, the data collected were deemed reliable. The average variance extracted (AVE) was subsequently calculated to test the convergence validity of the constructs. The minimum requirement for the AVE is 0.5 [74], and all the constructs in the study produced higher AVE scores than the requirement, with 0.592 being the lowest score. The square roots of the AVE values were then compared against the correlations of each pair to establish the discriminant validity. In general, all the correlations were lower than the square roots of the AVEs except the correlation between behavioral intentions and sensation seeking. Thus, the pair was subjected to a further step of testing by combining the items of both constructs into one. Then, a model comparison test was performed for the original measurement model vs. the measurement model with combined items. The result showed that both models were statistically different (∆*χ*^2^ = 110.045, ∆*df* = 7, *p* = < 0.001). Hence, evidence of discriminant validity existed. A summary of the CFA results can be seen in Table 1.

### 4.2. Structural Equation Modeling

After successfully validating the measurement model, the second step was to examine the relationships between the latent constructs as hypothesized. Similar to the first step, the model fit statistics of the structural model were first examined. The fit indices of the structural model were also satisfactory (*χ*^2^ = 382.427, *df* = 180, *χ*^2^/*df* = 2.125, RMSEA = 0.063, CFI = 0.957, IFI = 0.958, TLI = 0.950, NFI = 0.923, and PGFI = 0.688). Path analysis found that all but one of the proposed relationships were significant. Specifically, the path between involvement and knowledge was significant and positive (β = 0.581, *p* = < 0.001). Hence, Hypothesis 1 was supported by the data. Hypothesis 2, involvement–attitude (β = 0.461, *p* = < 0.001) and Hypothesis 3, involvement to perceived control (β = 0.540, *p* = < 0.001), were also supported. Hypothesis 4, knowledge to attitude (β = 0.199, *p* = < 0.01), and Hypothesis 5, knowledge to perceived control (β = 0.236, *p* = < 0.001), were both supported by the data. Subsequently, the influence of the three antecedents of the TPB on intentions found attitude (β = 0.392, *p* = < 0.001) and perceived control (β = 0.620, *p* = < 0.001) to be significant, but subjective norms did not yield a significant result (β = 0.019, *p* = > 0.05). The SEM results also showed that 75.2% of the total variance explained the final construct, intentions. Lastly, involvement was found to produce the largest total impact on intentions (0.646), followed by perceived control (0.620). Table 2 presents a summary of the SEM results.

**Figure 2:**
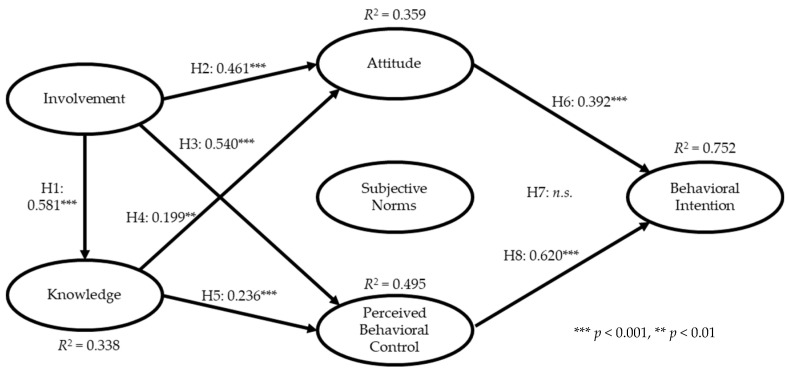
Conceptual model and structural equation modeling (SEM) results.

Another part of the SEM process was to test the indirect effects among the variables using the bootstrapping method. The number of bootstrap samples was 2000 at 95 percentile confidence intervals. The test was conducted to determine the type of mediating effect between two variables in the proposed model. The results found all the mediators to be partial mediators, hence, significant indirect effects among all the indirect relationships. Table 3 provides a summary of the indirect effect assessment. Additionally, the conceptual model with the SEM results is illustrated in Figure 2.

### 4.3. Multigroup Moderation

Hypotheses 9a–h were tested using the multigroup invariance test method. Hence, the method first categorized sensation seeking into high and low groups, determined the invariance at the measurement-model level, and tested the invariance of each path at the structural-model level. The study used k-means clustering to determine which research samples exhibited high (or low) levels of sensation seeking according to the measurement items used. The results grouped 255 samples into the high group (mean = 4.27) and 32 into the low group (mean = 2.84). Then, the model fit statistics of the nonrestricted measurement model were compared to those of the full-metric invariance version. The results of the model comparison show both groups to be statistically different (△χ^2^ (21) = 35.408, p = < 0.05). Statistically different relationships between the high and low groups at the structural level were subsequently tested for. For the structural model, paths that were not significant for both groups were removed to establish the baseline model fit statistics (χ^2^ = 697.772, *df* = 362, χ^2^/*df* = 1.928, RMSEA = 0.057, CFI = 0.926, TLI = 0.914, and IFI = 0.927). Only one path from subjective norms to intentions was not significant in both groups. Then, each nested model was calculated, and if the result for the model comparison against the baseline model was significant, the moderating effect of sensation seeking was confirmed.

The moderating impact of sensation seeking was found to be significant in four relationships—specifically, Hypothesis 9b, the path from involvement to attitude (△χ^2^ (1) = 3.905, *p* = < 0.05). Although both the high- and low-sensation-seeking groups showed positive and significant relationships, the low-sensation-seeking group exhibited a stronger influence of involvement on attitude. In other words, those who are low on sensation-seeking personality tend to spend more time researching and reading about adventure tours. This ultimately predicts a favorable attitude towards adventure tours as established by the SEM results. Furthermore, the relationship between involvement and perceived control was also significantly moderated by sensation seeking (△χ^2^ (1) = 9.040, *p* = < 0.01). The relationship between knowledge and perceived control was also significantly moderated by sensation seeking (△χ^2^ (1) = 9.500, *p* = < 0.01). However, the low group exhibited a negative but still significant relationship. In other words, among low sensation seekers, the knowledge of adventure tourists does not predict their perceived capability in participating in adventure tours. Lastly, the relationship between attitude and intentions was also moderated by the sensation-seeking variable. Thus, Hypotheses 9c,e,f were also accepted. A summary of the multigroup invariance test results for sensation seeking is provided in Table 4.

Similar to the multigroup invariance test for sensation seeking, the moderating effect of worry was analyzed with the same procedure. The k-means clustering results yielded 169 samples in the high-worry group (mean = 3.59) and 118 in the low-worry group (mean = 2.08). The measurement model was also significantly different between the high and low groups (△χ^2^ (21) = 25.554, *p* = < 0.05). The path from subjective norms to intentions was also not significant for both groups and was removed before establishing the baseline model. The baseline model fit of the structural model was established as follows: χ^2^ = 591.179, *df* = 362, χ^2^/*df* = 1.633, RMSEA = 0.047, CFI = 0.950, TLI = 0.942, and IFI = 0.951. 

**Figure 3:**
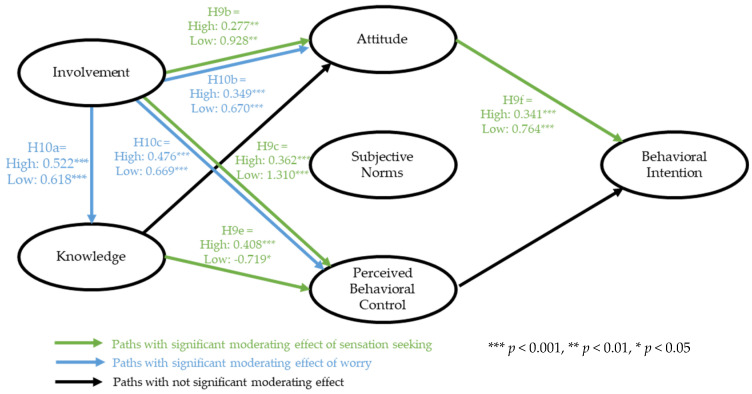
Conceptual model and multigroup invariance test results.

The path-by-path analysis found three relationships to be significantly moderated by worry. The path from involvement to knowledge was significant for both groups, with the low group having a slightly stronger effect (△χ^2^ (1) = 6.674, *p* = < 0.05). In other words, those who tended to worry less about adventure tours also showed a stronger prediction of a high level of involvement resulting in a higher level of knowledge. Similar to the first relationship, the paths from involvement to attitude (△χ^2^ (1) = 8.903, *p* = < 0.01) and to perceived control (△χ^2^ (1) = 3.321, *p* = < 0.10) were moderated by worry. The low-worry group also exhibited a stronger influence when compared to the high group. Hence, Hypotheses 10a–c were supported. Table 5 summarizes the results of the multigroup invariance test for worry. In addition, Figure 3 provides a graphical illustration of the moderating effects of both sensation seeking and worry on the conceptual model. 

## 5. Discussion

### 5.1. General Discussion

The proposed study model produced a robust predictive ability for the final construct, the behavioral intentions to participate in adventure tours, as 75.2% of the total variance explained the construct. The addition of both involvement and knowledge helped to improve the predictive ability based on their high total impact on intentions, 0.646 and 0.224, respectively. This suggests that those involved and knowledgeable about adventure tourism are likely to participate in the future. The results are consistent with previous findings on involvement and knowledge and their impact on intentions [40,53,55,56,70]. The successful extension of the TPB was also supported by the relatively strong model fit statistics, suggesting that the study model remained parsimonious even though additional constructs were added. Previous attempts at theory extensions also used such statistical results to determine the extended models’ efficacy [6,47,63,64,75,76].

The hypotheses were generally supported by the results. Individuals who spend time and effort to read and learn about adventure tourism are also likely to accumulate more knowledge about the activity, similar to previous findings [40,53,56]. Additionally, consistent with empirical evidence are the effects of involvement and knowledge on attitude and perceived behavioral control [38,39,42,43,56,65,70]. It is logical that individuals would spend time and effort to learn about and immerse themselves with things they are interested in. Furthermore, it is also expected that one would have a favorable attitude towards their own interests. Similarly, one would likely feel more confident engaging in familiar activities and those one has prior experience with. Still, involvement yielded a more substantial impact on attitude and perceived control than knowledge, suggesting that having prior experience and knowledge in the adventure tourism context is unnecessary. Thus, adventure tours are also suitable for a wider group of tourists but still not suitable for the general mass market. Hence, the additional constructs not only have statistical support but are also logically sound.

One of the three antecedents of the TPB, subjective norms, did not significantly predict behavioral intentions. Even though the three antecedents have often been found to consistently predict intentions, some past research projects did not find all three significant [6,49,58]. For example, in a food tourism context, subjective norms also did not significantly affect intentions [49]. The authors explained that due to the relatively novel and niche interests of food tourism, food tourists are less susceptible to external influences by their reference group. Similarly, in another adventure tourism study, the authors also found the subjective norms not to be a significant predictor of intentions [6]. In this study, the result was due to how adventure activities are not accepted by the public and how people are often recommended by close friends and family to avoid participating. Both lines of reasoning could also apply to this study. Adventure tourism tends to involve younger people and travel in groups with friends. The sample profiles of this study also supported this notion. At the same time, the roles of attitude and perceived behavioral control were important predictors of intentions as hypothesized and consistent with previous studies [6,49,60,62].

The moderating role of sensation seeking yielded some useful findings. Among the four paths, three were significantly moderated by sensation seeking; the path from knowledge to perceived control changed to negative among low sensation seekers while remaining significant. The relationship describes that if an individual has prior experience or knowledge about adventure tours, they also have the necessary skills and confidence to partake in adventure tours. However, those who have less tendency to seek new and arousing activities do not necessarily feel confident to participate even if they have prior experience. Similarly, the moderating impact of worry also shows that those with a high level of worry tend to rely on involvement more to feel confident and have control. The findings are consistent with previous studies in that those who are often worried tend to rely more on preparation and information as evidence to solve problems or make decisions [36,37]. In addition to these notable findings, the results suggest that high sensation seekers’ attitudes towards adventure are less influenced by their levels of involvement. This implies that attitudes can be either internally generated or driven by emotion rather than relying on a cognitive process. On the contrary, those in the low-worry group exhibited a stronger impact of involvement on knowledge and attitude. This supports the same notion as for those in the high-sensation-seeking group that adventure activities are not solely driven by a cognitive process but also a product of an individual emotional process.

### 5.2. Implications

The findings could be useful for various groups of stakeholders. In terms of practical implications, the study highlighted some key contributions. First, the adventure tourism subsector relies on a substantial amount of pre-trip information, such as descriptions about activities, preparation procedures, precautionary information, and knowledge sharing between members. Involvement has been proven to play a vital role within the sector. Adventure tour operators can leverage this nature by engaging more with the community through various online platforms. Operators should also focus on providing as much information to prospective tourists as possible, either on their websites or through social network groups. Unlike other tourism subgroups, adventure tourism does not necessarily benefit from elements of surprise. The excitement and thrill of the experience should already be stimulated by the natural settings and the physical requirements. Instead, the participants and tour operators should focus on the preparation and safety of all the people involved. Preparation and the information provided by the tour operators before the trip should create even more excitement. The more materials there are, the more participants would need to spend time learning and interacting. Hence, it would help to generate an even greater degree of involvement for the participants.

Adventure activities maintain the appeal of the risk and thrill elements. However, it is worth noting the difference between actual risk and perceived risk. While perceived risk can be leveraged as part of storytelling and marketing, actual risk should be avoided or minimized. The results also point out the critical role of knowledge and perceived control, which implies that the more one participates, the more experience and perceived control one accumulates. In combination with the varied level of expertise required for each of the adventure activities, it is imperative that the right person participates in the right type of activity and at the right level of difficulty. However, most destinations do not have a universal assessment and guidelines for adventure activities, making it difficult for beginners to find out where and what activity is most suitable for their skill level. Destinations or national tourism organizations may consider adopting universal classification systems such as those found among ski resorts (green/blue, red/black, and double black) to denote the level of difficulty. The classification would help to reassure those who tend to worry by giving them a higher level of certainty about the chosen challenge ahead. Simultaneously, gamification can help to motivate high-sensation-seeking individuals to challenge themselves further and achieve status among peers.

Theoretically, the study provided empirical evidence for the proposed study model, which adopted the well-established TPB and extended it with two additional critical variables. The new additional variables have been proven to be essential in explaining adventure tourism intentions, as they substantially improved the intentions’ explanatory power. The results for the two moderators also yielded satisfactory statistical scores as well as offering practical contributions. The sensation-seeking variable provided evidence on how adventure tourists are still fundamentally looking for emotional arousal that is not offered from other passive activities. At the same time, worry did not turn out to be a constraint or prohibit participation in adventure activities. Instead, it further highlighted the importance of pre-trip preparation. Both of the moderating variables have received little research attention in tourism research, although tourism activities involve many elements and varying degrees of uncertainty, which can be explained by both variables.

### 5.3. Limitations and Recommendations for Future Research

The study is bound by a few limitations. The study samples were limited to Vietnamese tourists. Thus, the social norms, types of activities, and difficulty levels are all strictly applicable to the Vietnamese adventure tourism context. However, other destinations may still find the results of this study appropriate and relevant. At the same time, future studies are encouraged, to replicate this study’s conceptual framework in other research contexts. The sample profiles are also limited in their generalizability’s scope, as the accurate distribution of the adventure tourists may not have been adequately captured in this study due to the data collection’s pragmatic restrictions. The findings of this study have also highlighted two avenues for future research. Firstly, the role of emotional arousal should be further explored. There are other types of emotional responses for adventure activities that other researchers can investigate, such as fear, escapism, stress relief, and more. Secondly, the critical role of pre-trip arrangement and preparation could be explored. The concept of worry is strongly linked to the uncertainty before the trip. Therefore, if tour operators can prepare the participants adequately in the pre-trip stage, many adventure activities could appeal to the mass market. Lastly, a study on the differences in emotion and cognition between those who have previous adventure tour experience and those who do not could yield insightful findings for tour operators.

## Figures and Tables

**Table 1:** Summary of confirmatory factor analysis results.

	INVO	KNOW	ATTI	NORM	CONT	INTEN	SENS	WORR
INVO	0.892 ^a^							
KNOW	0.574 ^b^	0.898						
ATTI	0.537	0.440	0.769					
NORM	0.345	0.397	0.454	0.784				
CONT	0.650	0.530	0.666	0.332	0.832			
INTEN	0.675	0.565	0.742	0.362	0.819	0.873		
SENS	0.577	0.402	0.677	0.210	0.726	0.792	0.782	
WORR	−0.132	−0.333	−0.183	−0.277	−0.224	−0.213	−0.094	0.860
AVE	0.795	0.806	0.592	0.614	0.692	0.763	0.611	0.740
CR	0.940	0.926	0.813	0.861	0.870	0.928	0.824	0.895

^a^ Squared root of AVE. ^b^ Correlation. **Note 1.** Goodness-of-fit statistics: *χ^2^* = 466.792, *df* = 296, *p* = < 0.001, *χ*^2^/*df* = 1.577, RMSEA = 0.045, CFI = 0.971, IFI = 0.971. **Note 2.** INVO = Involvement, KNOW = Knowledge, ATTI = Attitude, NORM = Subjective norms, CONT = Perceived control, INTEN = Behavioral intention, SENS = Sensation seeking, WORR = Worry.

**Table 2:** Summary of structural equation modeling results.

			Standardized Estimate	*t*-Value
**Hypothesis 1**: Involvement	→	Knowledge	0.581	10.164 ***
**Hypothesis 2**: Involvement	→	Attitude	0.461	5.896 ***
**Hypothesis 3**: Involvement	→	Perceived control	0.540	7.553 ***
**Hypothesis 4**: Knowledge	→	Attitude	0.199	2.713 **
**Hypothesis 5**: Knowledge	→	Perceived control	0.236	3.711 ***
**Hypothesis 6**: Attitude	→	Behavioral intentions	0.392	7.152 ***
**Hypothesis 7**: Subjective norms	→	Behavioral intentions	0.019	0.464
**Hypothesis 8**: Perceived control	→	Behavioral intentions	0.620	9.834 ***
**Goodness-of-fit statistics:***χ*^2^ = 382.427, *df* = 180, *χ*^2^/*df* = 2.125, RMSEA = 0.063, CFI = 0.957, IFI = 0.958, TLI = 0.950, NFI = 0.923, PGFI = 0.688 *** *p* < 0.001, ** *p* < 0.01		**Total variance explained:** *R*^2^ of INTEN = 0.752 *R*^2^ of CONT = 0.495 *R*^2^ of ATTI = 0.359 *R*^2^ of KNOW = 0.338	**Total impact on behavioral intentions:** INVO = 0.646 KNOW = 0.224 ATTI = 0.392 NORM = 0.019 CONT = 0.620	

Note: INVO = Involvement, KNOW = Knowledge, ATTI = Attitude, NORM = Subjective norms, CONT = Perceived control, INTEN = Behavioral intentions.

**Table 3:** Summary of indirect effect assessment results.

Indirect Effect of	On		
	ATTI	CONT	INTEN
**INVO**	0.116 **	0.137 **	0.646 **
**KNOW**	-	-	0.224 **

**Note 1.** *** *p* = < 0.001, ** *p =* < 0.01, * *p =* < 0.05. **Note 2.** INVO = Involvement, KNOW = Knowledge, ATTI = Attitude, CONT = Perceived control, INTEN = Behavioral intentions.

**Table 4:** Summary of multigroup invariance test results (sensation seeking).

**Sensation seeking—measurement-invariance model for high (*n* = 255) and low (*n* = 32)**										
**Models**			**χ^2^**		***df***		**△χ^2^**			**Full-metric invariance**
Nonrestricted model			608.681		348		△χ^2^ (21) = 35.408, *p* = < 0.05			Not supported
Full-metric invariance			644.089		369					
Model fit statistics for the nonrestricted model: RMSEA = 0.051, CFI = 0.942, TLI = 0.931, IFI = 0.943.										
Model fit statistics for the full-metric model: RMSEA = 0.051, CFI = 0.939, TLI = 0.931, IFI = 0.940.										
**Sensation seeking—structural-invariance model for high (*n* = 255) and low (*n* = 32)**										
**Paths**	**High**			**Low**				**Nested model**	**Chi-square difference test**	
	**β**	***t*-values**		**β**		***t*-values**		**(equally restricted)**		
**H9a:** IN → KN	0.586	9.454 ***		0.826		4.278 ***		χ^2^ (363) = 699.499	△χ^2^ (1) = 1.725, *p* = > 0.10	
**H9b:** IN → AT	0.277	3.247 **		0.928		2.907 **		χ^2^ (363) = 701.679	△χ^2^ (1) = 3.905, *p* = < 0.05	
**H9c:** IN → CO	0.362	4.866 ***		1.310		3.396 ***		χ^2^ (363) = 706.814	△χ^2^ (1) = 9.040, *p* = < 0.01	
**H9d:** KN → AT	0.326	3.800 ***		−0.359		−1.157		χ^2^ (363) = 699.843	△χ^2^ (1) = 2.069, *p* = < 0.10	
**H9e:** KN → CO	0.408	5.452 ***		−0.719		−2.140 *		χ^2^ (363) = 707.274	△χ^2^ (1) = 9.500, *p* = < 0.01	
**H9f:** AT → INT	0.341	5.343 ***		0.764		6.006 ***		χ^2^ (363) = 704.959	△χ^2^ (1) = 7.185, *p* = < 0.01	
**H9g:** NO → INT	0.018	0.369		0.076		1.146		-	-	
**H9h:** CO → INT	0.621	8.316 ***		0.327		3.084 **		χ^2^ (363) = 699.928	△χ^2^ (1) = 2.154, *p* = > 0.01	
Baseline model fit: χ^2^ = 697.772, *df* = 362, χ^2^/*df* = 1.928, RMSEA = 0.057, CFI = 0.926, TLI =0.914, IFI = 0.927.										

**Note 1.** *** *p* = < 0.001, ** *p =* < 0.01, * *p =* < 0.05. **Note 2.** IN = Involvement, KN = Knowledge, AT = Attitude, NO = Subjective norms, CO = Perceived control, INT = Behavioral intentions.

**Table 5:** Summary of multigroup invariance test results (worry).

**Worry—measurement-invariance model for high (*n* = 169) and low (*n* = 118)**										
**Models**			**χ^2^**		***df***		**△χ^2^**			**Full-metric invariance**
Nonrestricted model			507.661		348		△χ^2^ (21) = 25.554, *p* = < 0.05			Not supported
Full-metric invariance			533.215		369					
Model fit statistics for the nonrestricted model: RMSEA = 0.040, CFI = 0.965, TLI = 0.958, IFI = 0.966.										
Model fit statistics for the full-metric model: RMSEA = 0.040, CFI = 0.964, TLI = 0.959; IFI = 0.965.										
**Worry—structural-invariance model for high (*n* = 169) and low (*n* = 118)**										
**Paths**	**High**			**Low**				**Nested model**	**Chi-square difference test**	
	**β**	***t*-values**		**β**		***t*-values**		**(equally restricted)**		
**H10a:** IN → KN	0.522	6.680 ***		0.618		7.114 ***		χ^2^ (363) = 597.853	△χ^2^ (1) = 6.674, *p* = < 0.05	
**H10b:** IN → AT	0.349	3.507 ***		0.670		5.143 ***		χ^2^ (363) = 600.082	△χ^2^ (1) = 8.903, *p* = < 0.01	
H10c: IN → CO	0.476	7.649 ***		0.669		5.635 ***		χ^2^ (363) = 594.500	△χ^2^ (1) = 3.321, *p* = < 0.10	
**H10d:** KN → AT	0.213	2.098 *		0.047		0.439		χ^2^ (363) = 592.401	△χ^2^ (1) = 1.222, *p* = > 0.10	
**H10e:** KN → CO	0.249	2.406 *		0.118		1.297		χ^2^ (363) = 593.051	△χ^2^ (1) = 1.872, *p* = > 0.10	
**H10f:** AT → INT	0.379	5.167 ***		0.424		4.592 ***		χ^2^ (363) = 591.239	△χ^2^ (1) = 0.060, *p* = > 0.10	
**H10g:** NO → INT	0.019	0.300		0.018		0.288		-	-	
**H10h:** CO → INT	0.630	7.861 ***		0.559		5.658 ***		χ^2^ (363) = 591.291	△χ^2^ (1) = 0.112, *p* = > 0.10	
Baseline model fit: χ^2^ = 591.179, *df* = 362, χ^2^/*df* = 1.633, RMSEA = 0.047, CFI = 0.950, TLI =0.942, IFI = 0.951.										

**Note 1.** *** *p* = < 0.001, ** *p =* < 0.01, * *p =* < 0.05. **Note 2.** IN = Involvement, KN = Knowledge, AT = Attitude, NO = Subjective norms, CO = Perceived control, INT = Behavioral intentions.

## Data Availability

Not applicable.

## Appendix A

**Table A1:** Measurement items.

Constructs	Measurement Items
Knowledge	Compared to other people, I know a lot about hard adventure tourism. My friends consider me an expert regarding hard adventure tourism. I consider myself very experienced in the hard adventure tourism field.
Involvement	I read hard adventure trip reviews from other travelers. I searched for hard adventure travel information on social media websites. I looked at hard adventure activity/attraction reviews of other travelers. I read other hard adventure travelers’ experiences and trips.
Attitude	I think participating in a hard adventure tour is a positive behavior. I think participating in a hard adventure tour is a valuable behavior. I think participating in a hard adventure tour is a beneficial behavior. I think participating in a hard adventure tour is a necessary behavior.
Subjective norms	Most people who are important to me agree that I participate in hard adventure tours. Most people who are important to me support that I participate in hard adventure tours. Most people who are important to me understand that I participate in hard adventure tours. Most people who are important to me recommend that I participate in hard adventure tours.
Perceived behavioral control	I am confident that if I want, I can participate in a hard adventure tour. I am capable of joining a hard adventure tour. I have enough resources (e.g., money) to participate in a hardy tour.
Behavioral intentions	I will make an effort to participate in a hard adventure tour in the future. I have an intention to participate in a hard adventure tour. I am willing to participate in a hard adventure tour. I am willing to save time and money to participate in a hard adventure tour.
Sensation seeking	I would like to explore strange places. I would like to take a trip with no pre-planned routes or timetables. I get restless when I spend too much time at home. I prefer friends who are excitingly unpredictable. I like to do frightening things. I would like to try hard adventure tours. I like wild parties. I would love to have new and exciting experiences, even if they are illegal.
Worry	I constantly worry that something may go wrong when I participate in a hard adventure tour. I worry that I’ll get lost or lose contact with my travel companions when I participate in a hard adventure tour. I worry that hard adventure tour is dangerous and scary.
